# Closing the Gaps: competing estimates of Indigenous Australian life expectancy in the scientific literature

**DOI:** 10.1111/1753-6405.12084

**Published:** 2013-08

**Authors:** Amanda Rosenstock, Bryan Mukandi, Anthony B Zwi, Peter S Hill

**Affiliations:** Australian Centre for International and Tropical Health, School of Population HealthUniversity of Queensland; School of Social Sciences and International StudiesUniversity of New South Wales; Australian Centre for International and Tropical Health, School of Population HealthUniversity of Queensland

**Keywords:** Indigenous, Australian, Aboriginal and Torres Strait Islander, life expectancy, mortality, longevity

## Abstract

***Objective:*** Closing the gap in life expectancy between Indigenous and other Australians within a generation is central to national Indigenous reform policy (Closing the Gap). Over time, various methods of estimating Indigenous life expectancy and with that, the life expectancy gap, have been adopted with differing, albeit non-comparable results. We present data on the extent of the gap and elucidate the pattern of use and interpretations of the different estimates of the gap, between 2007 and 2012.

***Methods:*** An extensive search was conducted for all peer-reviewed health publications citing estimates of and/or discussing the life expectancy of Indigenous Australians, for the period 2007–2012.

***Results:*** Five predominant patterns of citation of the gap estimates were identified: 20 years, 17 years, 15–20 years, 13 years, and 11.5 years for males and 9.7 years for females. Some authors misinterpret the most recent estimates as reflecting improvement from the 17-year figure, rather than the result of different methods of estimation. Support for the direct methods used to calculate Indigenous life expectancy is indicated.

***Conclusions and Implications:*** A specific estimate of the life expectancy gap has not been established among stakeholders in Indigenous health. Agreement on the magnitude of the gap is arguably needed in order to evaluate strategies aimed at improving health outcomes for Indigenous Australians. Moreover, measuring progress towards ‘closing the gap’ depends on the availability of comparable estimates, using the same techniques of measurement to assess changes over time.

The health inequities between Indigenous (used here to denote the Aboriginal and Torres Strait Islander peoples of Australia) and non-Indigenous Australians are well documented.^1^–^4^ At a population level, Indigenous Australians experience greater morbidity, mortality and disability across multiple conditions and at every stage of the life course.^1^ For example, higher rates of diabetes, renal disease and impaired vision, disproportionate rates of hospitalisation for mental health problems, a greater incidence of intentional injury and higher rates of hospitalisation and death due to cardiovascular disease, affect Indigenous compared to non-Indigenous people.^1^ These disparities are attributable to the complex interplay of historical processes and a wide range of biological, socio-cultural, political and economic determinants of health.^5^,^6^ As in many settings, these inequities are a cause of resentment and dissatisfaction by those whose health is most affected, and this is of concern to policy makers motivated to promote social justice and a more equitable society.^7^ The comparatively poor reported health status of the Indigenous population is starkly reflected in the wide differentials in mortality rates relative to the non-Indigenous population.^8^

Life expectancy at birth is a summary measure of mortality and is commonly used as an indicator for social equity.^9^ In Australia, it has been used to assess the relative disadvantage of the Indigenous population,^8^,^10^,^11^ and has served as a powerful device in advocacy for Indigenous health. Its calculation, however, is dependent on the quality of information about population size and deaths by age and sex,^10^ which forms the basis of the estimation of the life expectancy of the Australian population by the standard or direct approach. Concern around the quality of Indigenous data, especially data on Indigenous deaths, has led to the use of various indirect methods of estimating Indigenous life expectancy.^12^ As a result, over time, various methods of estimating the life expectancy of Indigenous Australians – and with that the life expectancy ‘gap’– have been adopted, producing differing results (see Table 1).^13^–^17^

**Table 1:** Calculated average & sex-specific estimates of the life expectancy gap, method and source.

Average Estimate	Sex-specific Estimates	Method	Source
20 years^a^	18 years (males) & 19 years (females)	Preston-Hill (indirect)	Australian Bureau of Statistics (ABS) 1998
17 years	17 years (males) & 17 years (females)	Bhat (indirect)	ABS 2004
13 years	12.5 years (males) & 13.5 years (females)	Generalised Growth Balance (GGB) (indirect)	Vos et al. 2007 Hill et al. 2007^b^
−	11.5 years (males) & 9.7 years (females)	Direct	ABS 2009

a *In 2011, the ABS cited an estimate of ‘approximately 20 years’.10*

b *Hill et al.17 compare estimates of life expectancy at birth for the Indigenous population over the period 1996–2001, to estimates for the total Australian population from 1998–2000, whereas Vos et al.^16^ compare estimates only for the period 1996–2001. Hill et al.^17^ calculate life expectancy gaps of 12.4 years for males and 13.1 years for females*.

Following the citation of the 17-year gap in the *Social Justice Report 2005*,^18^ health and human rights advocates combined to establish the Close the Gap campaign for health equality.^19^ In 2007, Close the Gap succeeded in securing a commitment from the Council of Australian Governments (COAG), the peak body representing federal, state, and territorial cooperation on policy reform, to close the gap in life expectancy within a generation.^19^ The following year, the Indigenous Health Equality Summit brought forth a Statement of Intent to ‘close the gap in life expectancy by the year 2030’, citing the 17-year figure as its baseline.^20^ COAG later adopted this commitment as the first of six targets in the National Indigenous Reform Agreement (NIRA), also known as Closing the Gap.^21^ Child-specific targets included in the framework aim to reduce the gaps in mortality, literacy, numeracy and Year 12 attainment by 50%, as well as to ensure access to early childhood education for Indigenous four-year-olds in remote communities.^22^ Additionally, a commitment has been made to halve the gap in employment outcomes between Indigenous and other Australians.^22^

Since its introduction, Closing the Gap has been the principal guiding framework for the National Partnership Agreements (NPAs), which specify funding arrangements related to each of the targets, between the Federal and State and Territory Governments.^22^ Moreover, the annual *Closing the Gap Prime Minister’s Report*,^22^ the annual COAG *National Indigenous Reform Agreement: Performance Report*,^23^ the biennial Productivity Commission *Overcoming Indigenous Disadvantage Report*^4^ and the biennial Office for Aboriginal and Torres Strait Islander Health (OATSIH) *Aboriginal and Torres Strait Islander Health Performance Framework Report*^24^ all take improvement in estimated Indigenous life expectancy at birth to be a key performance indicator.

In Australia, an individual is officially recognised as being Indigenous if they identify themselves and are recognised by the community as being of Aboriginal or Torres Strait Islander descent.^25^,^26^ The election of individuals to self-identify in some instances and refrain from doing so in others, as well as systemic problems around ascertaining and recording Indigenous identity, affect the calculation of mortality rates and thus, life expectancy at birth.^27^ The increasing propensity of individuals to identify as Indigenous between population censuses also affects the calculation of life expectancy.^10^ Each method applied to the estimation of Indigenous life expectancy encompasses a unique set of assumptions, in order to adjust for bias in Indigenous death and population counts (Table 2).^10^ As a result, different estimates are non-comparable and cannot be interpreted as reflecting changes over time.^10^

**Table 2:** A brief description of the methods used to estimate Indigenous life expectancy.

Method	Description
Preston Hill (1980)^28^	Adjusts number of deaths according to population count over intercensal period Assumes stable population Does not account for interncensal changes in Indigenous identification
Bhat (2002)^29^	Includes ‘migration’ adjustment to correct for change in Indigenous identification (‘unexplained growth’) between censuses Assumes natural increase in population
Generalised Growth Balance (GGB) (1987)^30^	Assumes change in census coverage to account for change in Indigenous identification between censuses
ABS direct method (2008)^31^	Verifies Indigenous status by linking mortality records directly to census data

Despite the instability of the estimates, closing the gap in life expectancy between the Indigenous and non-Indigenous population remains a priority for Australian policy makers. Thus, it is necessary to gain a clearer understanding of how changes in the estimates have been considered within the policy, political and rhetorical context of the gap. This paper seeks to present data on the extent of the gap, as well as to elucidate both the pattern of use and interpretations of the various estimates of the Indigenous life expectancy gap between 2007 and 2012, across the peer-reviewed scientific literature.

## Aboriginal and Torres Strait Islander Life Expectancy Estimates 1998–2009

The Australian Bureau of Statistics (ABS), Australia’s national statistics agency, first published estimates of the Indigenous resident population in 1994.^32^ Due to the lack of reliable demographic data on the Indigenous population, estimates were technically considered to be ‘experimental’.^32^ In 1998, estimates of Indigenous life expectancy were published by the ABS, based on experimental life tables for the intercensal period 1991–1996.^15^ These life tables were constructed using the indirect demographic method proposed by Preston and Hill^28^ (Table 2), in order to adjust for under-identification of Indigenous status in death registrations relative to Indigenous population counts at each census.^15^ They produced Indigenous life expectancy estimates of 57 years for males and 62 years for females between 1991 and 1996. Subsequent estimates of 56 years for males and 62 years for females were calculated for the periods 1997–1999 and 1999–2001.^33^ By these estimates, Indigenous life expectancy was approximately 20 years lower, for both males and females, than that of the total Australian population.^33^

In 2004, the ABS produced life tables for the period 1996–2001 based on the indirect demographic method proposed by Bhat,^29^ recognising that its previous method questionably assumed a stable population and did not correct for intercensal changes in Indigenous identification (Table 2).^10^ A stable population occurs when constant age-specific fertility and mortality rates are applied to a population (no migration) over a sufficient period of time.^36^ The ABS has confirmed that this is inconsistent with the Australian Indigenous context.^35^ The new indirect method included a ‘migration’ adjustment to correct for the ‘unexplained growth’ of the Indigenous population due to intercensal changes in identification.^14^ Indigenous life expectancy estimates calculated using the Bhat method were 59 years for males and 65 years for females; this corresponds to a difference of approximately 17 years less compared to all Australians.^34^ Due to the differences in methodology used between the periods 1991–1996 and 1996–2001, the ABS indicated that the corresponding estimates cannot be compared or interpreted as reflecting changes in life expectancy over time.^35^

Different estimates for Indigenous life expectancy at birth for the period 1996–2001, based on the Generalised Growth Balance (GGB) method^30^ (Table 2), were published in *The Burden of Disease and Injury in Aboriginal and Torres Strait Islander Peoples 2003* study by Vos et al.^16^ In a review of the Bhat^29^ method used by the ABS, Vos et al.^16^ argued against the application of a migration adjustment given the negligible net migration among the Indigenous population. The GGB method is similar to the Bhat method; however, it assumes that the increasing propensity to identify as Indigenous is explained by differences in census coverage instead of migration.^16^ Indigenous life expectancy estimates for the period 1996–2001 were reported as 64 years for males and 69 years for females.^16^,^17^ This corresponds to a 13-year gap, for both males and females, between the Indigenous and total Australian population.^16^,^17^ An overview of the GGB method and the corresponding estimates were also published by Hill, Barker and Vos^17^ in 2007, challenging the dominant 17-year estimate for the gap between the Indigenous and total Australian population.^16^,^17^

Following the 2006 Census, the ABS proposed a direct demographic method for constructing Indigenous life tables (Table 2).^31^ The direct method involves linking death registrations to census data to ascertain coverage for Indigenous status, as opposed to relying on the assumptions required by indirect methods to correct for Indigenous under-identification in mortality records.^31^ On this basis, the ABS produced experimental Indigenous life expectancy estimates of 68 years for males and 74 years for females, for the period 2005–2007.^13^ These estimates correspond to 11.5 and 9.7 year gaps in life expectancy for males and females respectively, when compared to non-Indigenous males and females.^13^ However, due to incomplete Indigenous identification in death registrations, and the fact that 26% of registered Indigenous deaths could not be linked to a Census record, the ABS indicated that: “…these figures generally overestimate the true life expectancies”^31^ (p. 33). Again, the ABS warned that current and previous estimates are not comparable nor are differences indicative of trends in life expectancy over time.^13^

Although there is support for the application of direct methods^13^,^17^,^37^, the specific methodology used by the ABS to estimate Indigenous deaths has not gained broad agreement across those working in Indigenous health.^19^,^38^ The ABS substitutes Indigenous identification in death records for identification in census records, as opposed to the supplementary linkage of both data sources^38^, with Madden et al.^38^ arguing that this technique results in overestimates (upward bias) of life expectancy based on national, state and regional data.

## Literature Search Strategy

A search was undertaken for all peer-reviewed health publications citing Indigenous life expectancy estimates and/or discussing Indigenous life expectancy, within the period 2007 to 2012. This timeframe was chosen to allow for comparisons between citations corresponding to estimates published by the ABS and those calculated by Vos et al.^16^ and Hill et al.^17^

This search was limited to the peer-reviewed literature, despite the contribution of the community sector and the media to public perceptions of ‘the gap’. Howard^39^ suggests that peer review be thought of as ‘boundary work’, demarcating ‘science’ from ‘non-science’. While the distinction between ‘science’ and ‘non-science’ is subject to debate, the focus on peer-reviewed literature ensures the exclusion of sources that may have been dismissed out of hand, and provides the academic community an opportunity for self-reflection.

The following databases were used to retrieve the literature: Web of Knowledge – which includes literature available from the MEDLINE® database – Informit Complete, Embase and PubMed.

Key words/terms used in the Web of Knowledge and Informit searches included: [Indigenous Australian] OR [Aboriginal and Torres Strait Islander] AND [“life expectancy” OR longevity OR life span OR mortality] AND [health]. It was not necessary to narrow the searches in Embase and PubMed to health-specific literature, thus broader key words/terms were used: [Indigenous AND Australia*] OR [Aboriginal and Torres Strait Islander].

A total of 7,581 (including non-peer-reviewed) publications were retrieved from all searches. After discarding duplicated results and extraneous publications (unrelated to the subject of interest), this total was reduced to 2,039. Publications not specifically pertaining to the health of the Indigenous Australians were excluded, though studies comparing the health of Indigenous Australians to other Indigenous populations internationally were retained.

Following the application of these exclusion criteria, a total of 1,635 publications were manually searched for the terms: ‘expec*’, ‘longevity’, ‘liv*’ (ie. ‘live’, ‘living’, ‘lives’) and ‘life’. A total of 392 publications for the period 2007–2012 cited a specific estimate(s) and/or discuss Indigenous life expectancy. Non-peer-reviewed articles were discarded and a total of 364 peer-reviewed publications were retained.

## Results

**Figure 1:**
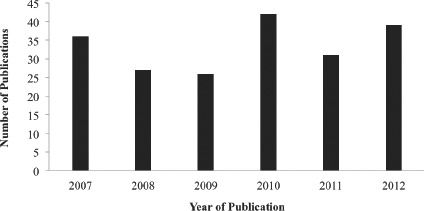
Publications citing estimates for Indigenous life expectancy*, 2007–2012.

Notes: * Includes estimates of the gap and estimates for absolute Indigenous life expectancy

Multiple estimates for the gap in life expectancy between Indigenous Australians and the Australian population are currently cited in the peer-reviewed scientific literature. A total of 201 out of the final 364 reviewed publications (55%) cited a specific Indigenous life expectancy estimate(s) or life expectancy gap. The number of publications citing specific estimates ranged from 26 to 42 (Figure 1).

**Figure 2:**
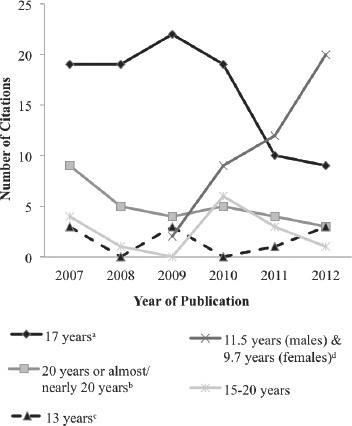
Estimates of the Indigenous life expectancy gap: citations* 2007–2012.

Notes: * Some authors cite more than one estimate in a single publication. *a Includes estimates of the gap reported as: 16–17 years, 17 years, 17–18 years and 18 years. Some authors*^40^–^44^*use the sex-specific differences (ie. 18 years for males and 17 years for females) in average life expectancy between the Indigenous and all Australian population, based on figures published in The health and welfare of Australia’s Aboriginal and Torres Strait Islander Peoples 2005.*^34^*However, overall (all persons) life expectancy is averaged as 17 years*.^34^*b Includes the estimate: ‘over 18 years’ cited by Hill et al.*^17^*based on life tables published by the ABS in 1998.*^15^*Also includes estimates of the gap reported as: ‘almost’, ‘nearly’, ‘about’ and/or ‘upto’ 20 yearsc Includes estimates of the gap reported as: 13 years*^16^,^17^, *12.5 years for males and 13.5 years for females16 and 12.4 years for males and 13.1 years for females*.^17^*d Includes estimates of the gap reported as: 11.5 years for males and 9.7 years for females, 10–12 years, 9–12 years, 11 years (high average) and 10 years (low average), based on figures published by the ABS in 2009.*^13^*Altman, Biddle and Hunter*^45^*and Biddle and Taylor*^46^*report the preliminary figures published by the ABS in 2008*,^31^*based on its revised methodology, as 11.8 years for females and 10.0 years for males*.

Following a review and comparison of citations between 2007 and 2012, five predominant patterns of citation of the gap estimates were identified, and are listed in chronological order: 20 years, or almost/nearly 20 years; 17 years; 15–20 years; 13 years; and 11.5 years for males and 9.7 years for females (Figure 2). Other estimated gaps are less frequently represented in the literature (Table 3).

**Table 3:** Authors citing other gaps: year of publication and gap cited.

Author(s)	Year of Publication	Gap Cited (years)
Boffa et al.	2007	17–20
Chan et al.^70^	2007	16–20
Cooke et al.	2007	23.2^a^
Calma	2010	10–17
Daniel at al.	2010	8–20^b^
Katzenellenbogen et al.	2010	13–17
Kruger et al.	2010a	17–20
Kruger et al.	2010b	17–20
Tsey et al.	2010	13–17
Daniel et al.	2011	8–20^b^
Brown et al.^71^	2012	10–20
Katzenellenbogen et al.^72^	2012	11–18
King et al.^73^	2012	17–19
Shepherd et al.	2012	11–14^c^

a *Cooke et al.^74^*^2007^ *report a gap of 23.2 years between Aboriginal and non-Aboriginal Australians*.

b *Daniel, Lekkas & Cargo75,76* *report an aggregate gap of 8–20 years between Aboriginal and non-Aboriginal populations internationally, by pooling estimates for Australia, Canada, New Zealand, and the United States*.

c *Shepherd, Li and Zubrick^77^*^2012^ *report a gap of 11–14 years between Australian Aboriginals and non-Aboriginals*.

The distribution of life expectancy estimates cited across the peer-reviewed literature for the period 2007–2012 is depicted in Figure 2. The variable citation of the estimates suggests that no single estimate of the gap has gained widespread acceptance, and all estimates are in current use. Despite the availability of the most recent figures published by the ABS in 2009, estimates produced prior to 2004 continue to be cited (Figure 2).

### The 20-year and almost/nearly 20-year gaps

The results shown in Figure 2 indicate that citations for the 20-year and almost/nearly 20-year estimates declined after 2007, but remain over subsequent years. Although more recent data are available, these estimates continued to be used in 2012.

### The 17-year gap

The main primary sources for citation of the 17-year estimate are the 2005^34^ and 2008^33^ editions of *The Health and Welfare of Australia’s Aboriginal and Torres Strait Islander Peoples*, published by the ABS and Australian Institute of Health and Welfare (AIHW). Both editions report the 17-year figure calculated by the ABS in 2004, based on the application of the Bhat method.^14^ The 17-year gap in life expectancy is the most widely cited estimate over the period of time investigated. However, as Figure 2 indicates, its use peaked in 2009, followed by decline in 2010. There nevertheless remains a persisting acceptance of its validity as a measure of health status for the Indigenous population, demonstrated in part by the fact that it was only beginning in 2011 that the most recent ABS estimates were cited more frequently.

### The 15–20 year gap

A total of 15 citations corresponding to a 15–20 year gap in life expectancy occur in the literature (Figure 2). The use of this estimate is problematic, as it appears to pertain to results for the Northern Territory published by Zhao and Dempsey;^47^ however, this could not be confirmed by a follow-up of referencing in all cases. Zhao and Dempsey^47^ performed a decomposition analysis of the causes of death contributing to the life expectancy gap between Indigenous and non-Indigenous Australians in the Northern Territory, from 1981 to 2000. The authors reported 95% confidence intervals for differences in life expectancies of 15.7 to 17.6 years for males and 18.1 to 20.0 years for females, for the period 1996–2000.^47^ Only two publications^48^,^49^ cite these findings directly for their reference to a 15–20 year estimate. All other citations for this gap are incorrectly referenced^50^–^61^ or do not provide a reference.^62^

### The 13-year gap

The corresponding pairs of gaps reported for males and females, respectively, as 12.5 and 13.5 years by Vos et al.^16^ and 12.4 and 13.1 years by Hill et al.^17^, based on the application of the GGB method, are included in this category. Compared to the ABS estimates, there has been little uptake of these estimates, with only 10 citations having been identified in this study. There has also been little attention paid to differences between the Bhat and GGB methodologies employed, respectively, by the ABS and both Vos et al.^16^ and Hill et al.^17^– despite the authors’ assertion that their estimates pose a direct challenge to the dominant perception of the gap.^16^,^17^

### The 11.5-year (male) and 9.7-year (female) gaps

A rise in the number of citations for the 11.5-year male and 9.7-year female gaps in life expectancy, published by the ABS in 2009,^13^ and based on direct methods, appears to coincide with a decline in the use of the 17-year estimate (Figure 2). The number of citations corresponding to the most recent ABS estimates increased from only two in 2009 to a maximum of 20 in 2012 (Figure 2). By 2012, the 11.5 and 9.7-year gaps accounted for almost half (48%) of all citations.

### Other gaps

A total of 14 citations corresponding to other gaps, excluding regional estimates, are presented in Table 3. The use of unique ranges in the literature warrants further attention. For example, several authors report a life expectancy gap of 17–20 years.^63^–^65^ Two publications^66^,^67^ cite a range of 13–17 years based on the estimates of Vos et al.,^16^ Hill et al.^17^ and the AIHW.^68^ Calma^69^ states that the Close the Gap campaign uses a gap of “10–17 years” to reflect the uncertainty associated with the change from the 17-year to 11.5 and 9.7-year estimates.

## Discussion

The variability in citations for the life expectancy gap in the literature suggests that no specific estimate has achieved universal recognition among stakeholders in Indigenous health. This is significant, given the stated aims of the Close the Gap campaign and COAG to close the life expectancy gap within a generation. While COAG has adopted the 2009 ABS figures as measures of the current gaps to be closed,^78^ other important stakeholders are working with different figures.

The limited use of the 20-year estimate is consistent with the timing of the establishment of Close the Gap which, by 2007, had propelled the 17-year estimate into the Australian public’s consciousness.^79^,^80^ However, there has been little response in the literature regarding the change in methodology underlying the transition from the 20 to 17-year estimate. Hill et al.^17^ impute an historical lack of concern for the mortality differentials of Indigenous populations globally to the absence of adjustment for bias, arising from variable reporting of Indigenous status in Indigenous death and population counts. This is consistent with the lack of correction for intercensal changes in Indigenous identification, based on the use of the Preston-Hill method prior to 2001.^35^ Thus, the low emphasis on the 20-year estimate in the literature may also reflect greater caution with respect to methodological uncertainty in the past. However, the rationale for the continued citation of the 20-year and almost/nearly 20-year estimates (Figure 2) remains unclear.

Compared to other estimates, the greater frequency of citations for the 17-year estimate suggests a persisting acceptance of its rhetorical face-validity in representing the comparative health status of the Indigenous population. The 17-year gap is commonly used to introduce the health disparities faced by the Aboriginal and Torres Strait Islander Australians,^37^,^79^,^81^,^82^ and reflects, in part, the influence of the Close the Gap campaign’s political advocacy and its subsequent impact in framing Indigenous health in Australia.

The limited use of the 13-year estimate in the literature may be attributable to its apparent challenge to the 17-year estimate, and concerns around the political impact of a lower, though not comparable estimate.^16^ This has implications for the acceptance of authority in the production and dissemination of data. Altman et al.^45^ and Cooke et al.^74^ acknowledge the calculation of the 13-year figure; however, they then rely on ABS estimates in their analyses of trends in Indigenous life expectancy over time.

While the use of the current COAG Closing the Gap baseline measure of the life expectancy gap (11.5 and 9.7 years) appears to be increasing, other estimates still account for more than half (52%) of all citations in 2012. Durey and Thompson^83^ claim that a gap of 11.5 and 9.7 years for men and women respectively is “… considered by many to be low, and subject to bias.” This may explain why these estimates have not gained greater traction in the literature. However, Durey and Thompson’s assessments are based on the results of two studies^84^,^85^ from Western Australia, in which the linkage of administrative data sets revealed under-identification of Indigenous and Aboriginal status, respectively, in mortality records. Thus, it is difficult to ascertain the implications of these findings for national estimates.

The representation of the gap as a range from estimates derived using different and non-comparable methodologies may compromise quantitative validity. However, the use of ranges in the literature draws attention to the problematic uncertainty resulting from a lack of comparability between estimates of Indigenous life expectancy.

### Interpretations of the changing estimates

The new ABS estimates have generated some confusion, given the rhetorical importance of the 17-year estimate to Indigenous health discourse in Australia. Despite ABS warnings against comparing different estimates of life expectancy for the Indigenous population, several authors misinterpret the change from previous estimates to those published in 2009 as evidence for improvement in the gap. For example, Tibby, Corpus and Walters^86^ argue that the 11.5 and 9.7-year figures suggest some improvement in estimates of the gap, despite the dominant perception of larger differentials between the Indigenous and non-Indigenous population. Moreover, Cerasa^87^ reports a narrowing of the life expectancy gap based on an incorrect interpretation of the current estimates. On the same basis, Arabena and Moodie^88^ claim that the simultaneous improvement of both Indigenous life expectancy, as demonstrated by the most recent estimates, and also life expectancy for other Australians, undermine the potential of an agreed target to close the gap within a generation.

In response to Tait’s interpretation^89^ of a narrowing in the gap from 17 years to 11.5 years for males and 9.7 years for females, McIver^90^ cautions against the failure to attribute the “apparent recent improvement” to methodological changes between the 2001 and 2006 censuses. McIver^90^ further draws attention to the ABS warnings and emphasises the need for accuracy in data management, in order to understand the impacts of efforts aimed at reducing the ‘true’ gap in life expectancy. Campbell^91^ cites the 11.5-year and 9.7-year estimates, but attributes the appearance of a ‘longer life expectancy’ compared to previous ABS estimates to changes in methodology.

In a critique of the ways in which Indigenous health issues are framed, McMurray^92^ argues that the emphasis on ‘closing the gap’ in Indigenous policy rhetoric is problematic, as it assumes the availability of specific interventions to achieve equality in health and life expectancy between the Indigenous and non-Indigenous populations. The author notes the inherent challenge of measuring the life expectancy gap, due to changes in the ABS’ methodology giving rise to the 17-year and subsequent 11-year (averaged) estimates.^92^ McMurrary^92^ further cautions against the interpretation of ‘any real reduction’, noting that the inability to accurately define and consequently measure changes in the gap hinder the evaluation of initiatives aimed at reducing health disadvantage in the Indigenous population.

### Support for the direct methods

There is some indication in the literature of a shift towards support for the direct methods employed by the ABS. Data quality, however, remains a problematic issue. While progress has been made with respect to data on the size of the Indigenous population, Indigenous deaths are not always recorded as such. As a result, Aboriginal and Torres Strait Islander deaths are underestimated.^12^,^31^ This fundamental question of data quality underlies the preponderance of methodological approaches to the estimation of Indigenous life expectancy. Even among those who welcome the use of direct methods such as Madden et al., the lack of accurate data raises methodological questions around how to best correct for those deficiencies.^38^

Cotter et al.^93^ challenge the idea that Indigenous Australians experience the same life expectancy and disability at age 50 as non-Indigenous people at age 70. They argue that the ‘iconic’ life expectancy gap of “nearly 20 years” as a component of policy rationale is not empirically justified.^93^ The authors emphasise that refinements in methodology and the current average estimate of 11 years challenge the existing perception of the gap.^93^ They use ABS life tables for the period 2005–2007 to analyse whether Indigenous people experience the same life expectancy as non-Indigenous people at age 70.^93^ Cotter et al.^93^ indicate that life expectancies for Indigenous males and females, at ages 65 and 63 respectively, are equal to those for the non-Indigenous population at age 70 – a gap of approximately 6 years – concluding that the current benchmark used in the planning of aged care services for the Indigenous population is inconsistent with both the 11-year and 6-year estimates.

Cotter et al.’s reliance on the 2009 life tables suggests an acceptance of the current methodology used to estimate Indigenous life expectancy, based on the perceived improvement from previous methods. This is consistent with the ABS^13^ assertion that the use of direct methods has garnered general support. However, it is important to note the ambiguity in the authors’ representation of the gap, as they neither cite nor provide a direct reference to the figure of “nearly 20 years”. It follows that the possible augmentation of the 17-year estimate to 20 years to inform aged care policy is also of concern. Interestingly, the authors question whether their findings imply a need for policy reform or a change in rhetoric surrounding the corresponding rationale.^93^ The inability to compare estimates and, therefore, measure changes in the gap over time, compromise the utility of the gap as guide for policy formulation and implementation. This is supported by Cotter et al.’s 93 conclusion that the current planning and delivery of aged care services to the Indigenous population may fail to adequately address the health needs of people in middle age groups.

McDermott expresses concern over the poor health status of Indigenous Australians relative to Indigenous populations internationally: “Where other OECD countries appear to have narrowed their Indigenous health gap over past decades, Australia seems uniquely unable to achieve this, and still reports a 17-year difference in life expectancy.”^37^ In a discussion of the findings from *The Burden of Disease and Injury in Aboriginal and Torres Strait Islander Peoples 2003*,^16^ McDermott^37^ also notes the 13-year gap in life expectancy published by Vos et al.,^16^ contrasting the GGB method’s adjustments for intercensal changes in Indigenous identification against previous indirect methods applied to calculations of the 17 and 20-year estimates.^37^ Moreover, McDermott^37^ argues that the direct methods employed by the ABS to produce the 11-year estimate demonstrate greater completion of Indigenous identification in mortality records and are better supported by empirical evidence, compared to the indirect methods. She subsequently cites the revised estimate of 11 years.^37^ While McDermott’s support for the direct methods is clearly indicated, it is inconsistent with her initial use of the 17-year estimate. This may be an oversight; however, it gives rise to confusion given the emphasis placed on the methodological improvements underlying the calculation of the new estimate.

Hill et al.^17^ and Draper et al.^84^ also recommend the use of direct methods in order to eliminate bias in census and death records due to Indigenous under-identification. Draper et al.^84^ investigate the impact of missing Indigenous status on mortality statistics in Western Australia, reporting reductions in life expectancy gains for both males and females previously calculated for the period 1997–2002, following the linkage of death records un-coded for Indigenous status to three alternate datasets. The authors conclude that the ascertainment of Indigenous identification has the potential to reveal less improvement in life expectancy than previously indicated.^84^ However, trends cannot be determined for the entire Indigenous population since direct methods have not been used to derive Indigenous life tables prior to 2005.^13^ The authors further affirm the importance of accounting for Indigenous under-identification in order to ensure that outcomes for the target to close the gap in life expectancy are not confounded by bias during data collection.^84^

While there is an overall paucity of responses to the use of direct methods, the increasing frequency of citations for the revised estimates (Figure 2) has implications for the interpretation of changes in the life expectancy gap. Future comparisons between estimates for the Indigenous population may be feasible if the current methodology used to calculate life expectancy remains consistent. Only then will it be possible to establish if real trends in the gap have occurred. Moreover, an accurate understanding of the gap may serve to better inform the evaluation of current policy objectives for improving the health of the Indigenous population.

## Conclusion and implications

The failure to identify a specific estimate of the gap in life expectancy that has gained acceptance among stakeholders in Indigenous health is largely due to changes in methodology, which have precluded comparisons between available estimates. Use of the changing estimates of the gap has not always been accompanied by a clear interpretation of corresponding methodological differences, suggesting that choice of estimate is influenced by more than just methodological stance or technical preferences.

In order to assess the impact of Closing the Gap on the health of the Indigenous population in Australia, changes in the gap must be measurable over time. Moreover, this requires confirmation of the current size of the gap to be closed. Yet, agreement as to the precise estimate of the gap has yet to be established. As former Aboriginal and Torres Strait Islander Social Justice Commissioner and Chair of the Close the Gap Steering Committee, Tom Calma, states: “…other than acknowledging that it exists we cannot say precisely how wide it is, apart from that it is large – at least 10 years large!”^69^

The inability to ascertain the magnitude of ‘the gap’ stands at odds with the policy target of closing that gap, thereby limiting the evaluation of strategies aimed at improving health outcomes for Indigenous Australians. However, the release of a new set of life expectancy estimates in 2013–2014, based on the continuing application of direct methods, is promising. It may allow for comparisons to be made with current data.^23^ That would be a significant step forward.

## Funding

This work formed part of the *Uptake of evidence to policy: the Indigenous Burden of Disease case study*, and was supported by the National Health and Medical Research Council [APP1010534].
